# Nasal Carriage by *Staphylococcus aureus* among Healthcare Workers and Students Attending a University Hospital in Southern Brazil: Prevalence, Phenotypic, and Molecular Characteristics

**DOI:** 10.1155/2020/3808036

**Published:** 2020-12-03

**Authors:** Tiago Danelli, Felipe Crepaldi Duarte, Thilara Alessandra de Oliveira, Raquel Soares da Silva, Daniela Frizon Alfieri, Guilherme Bartolomeu Gonçalves, Caio Ferreira de Oliveira, Eliandro Reis Tavares, Lucy Megumi Yamauchi, Marcia Regina Eches Perugini, Sueli Fumie Yamada-Ogatta

**Affiliations:** ^1^Programa de Pós-graduação em Fisiopatologia Clínica e Laboratorial, Universidade Estadual de Londrina-Rodovia Celso Garcia Cid, PR 445 km 380, Campus Universitário, Londrina, Paraná, Brazil; ^2^Departamento de Patologia, Análises Clínicas e Toxicológicas, Centro de Ciências da Saúde, Universidade Estadual de Londrina, Londrina, Brazil; ^3^Programa de Pós-graduação em Microbiologia, Universidade Estadual de Londrina, Londrina, Brazil; ^4^Departamento de Microbiologia, Centro de Ciências Biológicas, Universidade Estadual de Londrina, Londrina, Brazil; ^5^Programa Nacional de Pós-Doutorado-CAPES, Universidade Estadual de Londrina, Londrina, Brazil

## Abstract

**Background:**

*Staphylococcus aureus* can asymptomatically colonize the human anterior nares and skin, and nasal colonization by this bacterium represents a potential risk for development of invasive infections. The aim of this study was to determine the prevalence of *S. aureus* nasal carriage among healthcare workers and students attending a university hospital and to characterize the isolates phenotypically and molecularly.

**Methods:**

A cross-sectional study was performed with 324 volunteers. Cultures from nasal samples were obtained and *S. aureus* isolates were characterized according to their antimicrobial susceptibility profile and four virulence factors-encoding genes. MRSA isolates were characterized regarding their oxacillin/cefoxitin susceptibility, SCC*mec,* and REP-PCR types. Potential risks for *S. aureus* and MRSA carriage were analyzed.

**Results:**

Of 324 nasal samples, 42.9% were identified as *S. aureus*, of which 28.8% were MRSA. *S. aureus* carriers were significantly higher in males and students (OR = 2.898, 95%CI 1.553–5.410); however, no variables were associated with MRSA carriage. All isolates were susceptible to vancomycin and the highest rate of resistance was observed for penicillin (90.6%). All isolates harbored the *coa* gene, and 97.8%, the *icaA* gene; 15.8% and 6.5% were positive for *tst* and *lukS-PV/lukF-PV* genes, respectively. Among MRSA isolates, 45% carried the *mecA* gene but were phenotypically susceptible to oxacillin/cefoxitin; two harbored the *tst* and none had *lukS-PV/lukF-PV* genes. All MRSAs were distributed into six SCC*mec* types and type I (62.5%) was the most frequent. REP-PCR typing identified four main clusters among MRSA isolates.

**Conclusion:**

High prevalence of healthcare workers and students were identified as nasal carriers of *S. aureus* exhibiting different antimicrobial resistance profiles, including *mecA*-positive oxacillin-susceptible *S. aureus* (OS-MRSA) and the presence of virulence-encoding genes. Both cohorts may represent potential sources for the emergence of a successful *S. aureus* strain highly adapted to the hospital environment.

## 1. Introduction

*Staphylococcus aureus* can interact with its host as a commensal member of the microbiota [1] or as an opportunistic pathogen causing a wide range of community and hospital-associated infections [2–7]. In humans, this bacterium can colonize multiple body sites, but the nose (anterior nares) is the frequent ecological niche of *S. aureus* carriage. Indeed, around 20–30% of the human population can harbor persistently and asymptomatically this bacterium in this niche [1]. Importantly, persistent nasal colonization by *S. aureus* may increase the risk for subsequent infections and this scenario is even more complicated in hospitalized and immunocompromised individuals who can develop invasive infections with high morbidity and mortality rates [8, 9].

Nasal colonization by *S. aureus* may begin within the first days of human life and the horizontal transmission from colonized mother seems to be the major source of *S. aureus* carriage in newborns [10]. After birth, hands are the main vector for transmitting *S. aureus* from the surfaces to the nose [11, 12]. Particularly in hospitals, healthcare workers and patients who are nasal carriers may be the source for the transmission and spread of *S. aureus* in these settings [13–18]. Similarly, students who have continuous hospital exposure during their internship may represent an important source for transmitting *S. aureus* within the hospital environments [14, 19]. Crucially, high rates of colonization by methicillin-resistant *S. aureus* (MRSA) have been reported among healthcare workers [16, 17], and increasing rates of MRSA colonization among students with continuous hospital exposure have also been reported [19]. Besides their role in *S. aureus* transmission in hospital settings, healthcare workers and students may also serve as the vector for cross-transmission of MRSA between the community and hospital populations [14, 18].

Altogether these data indicate the importance of screening *S. aureus* nasal carriers in both healthcare workers and students as a component of the control of *S. aureus* infections in hospitals. In this regard, few studies on the prevalence of *S. aureus* colonization in healthcare workers and students attending the Brazilian hospital environments were reported in the literature.

The University Hospital of Universidade Estadual de Londrina (UH-UEL) is a public teaching hospital and the reference in high complexity cases in the north of Paraná, Brazil. The hospital serves as an internship field for undergraduate students of Medicine, Pharmacy, Nursing, and Physiotherapy and offers postgraduate programs in different biomedical skills. In a retrospective study performed during 2001–2015, MRSA accounted for 43.7% of bacteremia in hospitalized patients [5]. However, no study on nasal colonization by *S. aureus* of healthcare workers and students attending the hospital was performed so far. Therefore, the aims of this study were to estimate the prevalence of and the potential risk for *S. aureus* nasal carriage among healthcare workers and students of the UH-UEL and to characterize the isolates by phenotypic and molecular methods.

## 2. Materials and Methods

### 2.1. Study Design

A cross-sectional study was carried out with the community of the UH-UEL from December 2017 to May 2018. The sample size was calculated using the software EPI Info™ 7 (Atlanta, USA) and was based on the population (students and professionals) attending the UH-UEL, which amounts about 2000 individuals during the period of the study. Assuming a prevalence of *S. aureus* colonization of about 40% [20] and a confidence interval of 95%, at least 324 individuals were included in this study. The collection of data occurred at the time of nasal swab sampling through a standardized questionnaire that contained demographic variables, antibiotic use and/or hospitalization in the last three months, and direct contact with hospitalized patients. The study protocol was approved by the Ethics Committee of the UEL (CAAE 79663417.2.0000.5231-opinion number 2.421.361-CEP-UEL).

### 2.2. Collection and Processing of Nasal Swabs

One nasal sample was collected from each participant using the Stuart collection device (COPAN Diagnostic, Italy). The swab was introduced into a nostril and gently rotated three times, transported to the laboratory, and processed up to 2 h after collection. Nasal samples were inoculated into Tryptone Soya Broth (TSB, Oxoid, Brazil) supplemented with 6.5% sodium chloride and incubated at 35°C for 24 h. After, the sample was subcultured on Mannitol Salt Agar (Oxoid, Brazil) at 35°C for 24 h. Suggestive colonies of staphylococci were subjected to standard phenotypic identification [21]. Species identification was confirmed by a multiplex-PCR targeting the *nuc* gene (encoding thermonuclease) [22]. Bacteria were stored at −80°C in TSB containing 30% glycerol.

### 2.3. Antimicrobial Susceptibility Testing

The antibacterial susceptibility profiles for 12 antimicrobials were evaluated by the disk diffusion method. The minimal inhibitory concentration (MIC) for oxacillin was determined by the broth microdilution assay. The inducible clindamycin resistance was determined by the double disk diffusion method. All methods were performed as recommended by the Clinical and Laboratory Standards Institute [23]. The susceptibility breakpoints were those recommended by CLSI [23] except tigecycline that was interpreted according to the European Committee on Antimicrobial Susceptibility Testing [24]. *Enterococcus faecalis* ATCC 29212 and *S. aureus* ATCC 25923 were used as quality controls. Cefoxitin disk and oxacillin MIC were used to define MRSA.

To detect heterogeneous vancomycin-intermediate *S. aureus* (hVISA), 10 *µ*L of bacterial suspension (1.0–2.0 × 10^8^ CFU/mL) was spotted in duplicate onto Brain Heart Infusion agar supplemented with 3 *µ*g/mL and 4 *µ*g/mL vancomycin [25]. The systems were incubated at 35°C and the results were recorded at 24 and 48 h of incubation. *S. aureus* ATCC 29213 (MSSA), *S. aureus* BEC 9393 (MRSA), *S. aureus* Mu_3_ ATCC 700698 (hVISA), *S. aureus* Mu_50_ ATCC 700699 (VISA), and *E. faecalis* ATCC 51299 (VRE) were included as quality controls.

### 2.4. DNA Extraction

A single colony of each bacterium was cultured in 3 mL TSB at 35°C for 24 h. The cells were harvested by centrifugation (10,000 × g for 5 min), washed once with sterile 0.15 M phosphate-buffered saline pH 7.2, and resuspended in 300 *μ*L sterile solution (10 mM Tris-HCl, 1 mM EDTA pH8.0, and 1.0 mg/mL lysozyme). Genomic DNA was extracted as described previously [26], and 2 *μ*L were used in all amplification reactions.

### 2.5. Detection of Genes Encoding mecA and Virulence Factors by PCR

The gene *mecA* (encoding PBP2a) was detected as described by Milheiriço et al. [27]; the genes *icaA* (encoding *N*-acetylglucosaminyltransferase of the intercellular adhesion *locus*), *lukS-PV*/*lukF-PV* (encoding Panton-Valentine leukocidin-PVL), and *tst* (encoding toxic shock syndrome toxin-TSST-1) were detected as described by Campbell et al. [28], and the gene *coa* was detected as described by Tiwari et al. [29]. *S. aureus* BEC 9393 (*mecA*^+^, *icaA*^+^, *tst*^+^) and *S. aureus* ATCC 25923 (*coa*^+^, *lukS-PV*^+^/*lukF-PV*^+^) were used as controls.

### 2.6. MRSA Typing

SCC*mec* typing was performed by multiplex-PCR assay as described by Milheiriço et al. [27]. Nontypeable isolates were designated NT. *S. aureus* NCTC10442 (type I), N315 (type II), 85/2082 (type III), 81/108 (type IV), WIS [WBG8318] (type V), and HDE288 (type VI) strains were used as controls.

The genetic relatedness of all MRSA was analyzed by repetitive element sequence based-PCR (REP-PCR) [30]. Fingerprintings that had more than one band differing in size were considered different REP-PCR types [31]. Banding patterns were categorized using the UPGMA algorithm and Jaccard coefficient [32] of the Bionumerics v.6.5 software (Applied Mathematics, Kortrijk, Belgium), with the band tolerance set at 3% and the threshold cutoff value set at 85% [3].

### 2.7. Statistical Analyses

Analyses of contingency tables (*χ*^2^ test) were employed to check the associations between categorical variables and diagnostic groups. The Kolmogorov–Smirnov test was used to assess the normality of distribution of age. Categorical variables were expressed as absolute number (*n*) and percentage (%) and continuous variables were expressed as mean ± standard deviation. The association between explanatory variables and colonization was evaluated using three different models of automatic stepwise binary logistic regression analysis controlled for covariates that may confound the association of interest. All statistical analyses were performed using IBM SPSS windows version 20. Tests were 2-tailed and an alpha level of 0.05 indicated statistically significant results.

## 3. Results and Discussion

### 3.1. Baseline Characteristics of Participants and Prevalence of Nasal Carriage with *S. aureus* and MRSA

A total of 324 volunteers were enrolled in this study, where 103 (31.8%) were healthcare workers, 128 (39.5%) undergraduate, and 93 (28.7%) postgraduate students. The mean age of all participants was 30 ± 11.2 years ranging from 19 to 73 years, where 88 (27.2%) were males and 236 (72.8%) were females.

**Figure 1:**
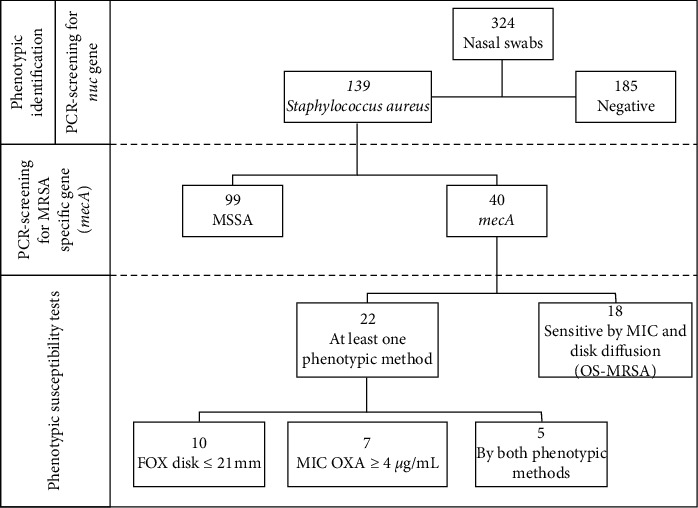
Flowchart of the procedures and the results for nasal swabs obtained from healthcare workers and students attending the University Hospital of Londrina from December 2017 to May 2018. PCR: polymerase-chain reaction; MSSA: methicillin-susceptible *Staphylococcus aureus*; MRSA: methicillin-resistant *S. aureus*; MIC: minimal inhibitory concentration; OS-MRSA: *mec*A-positive oxacillin-susceptible *S. aureus*; FOX: cefoxitin; OXA: oxacillin.

Although one single nasal sampling was performed on each participant, which does not allow the identification of persistent carriers [33], a high prevalence of *S. aureus* carriers was detected in this study. The overall prevalence of *S. aureus* nasal carriage was 42.9% (139/324); among them, 28.8% (40/139) were identified as MRSA carriers; that is, the isolate harbored the *mecA* gene, representing 12.3% of all participants (Figure 1).

### 3.2. *S. aureus* Nasal Carriage Was Significantly Higher in Males and Students

Colonization by *S. aureus* depends on bacterial ability to survive and adapt in the host's niches. These include overcoming innate and adaptive defenses of the host along with coexisting with other members of the microbiota that may promote or inhibit its growth [1]. Moreover, several factors including younger age, being male, contact with healthcare workers, professional occupation, and geographical region have been correlated with *S. aureus* nasal colonization [13, 34–36].

In this study, we analyzed six descriptive characteristics of the participants (gender, age, occupation, antibiotic use and/or hospitalization in the last three months, and direct contact with hospitalized patients) and their potential risk for *S. aureus* nasal carriage (Table 1). In order to evaluate the independence of associations, we have carried out three different automatic stepwise logistic regression analyses. In model #1 we added gender and occupation, both variables were positively associated with colonization with *S. aureus* (*p*=0.004 and *p*=0.019, respectively) (*χ*^2^ = 12.337, d*f* = 2, *p*=0.002). The second model (#2) is the model #1 added to the other explanatory variables evaluated in this study. Gender (male) remained associated with colonization (*p*=0.003), whereas age was negatively associated with colonization (*p*=0.021) (*χ*2 = 12.915, d*f* = 2, *p*=0.002). Finally, in model #3, when evaluating the interaction between gender (male) and occupation (student), only students of male gender were independently associated with colonization by *S. aureus*(*p*=0.001) (*χ*2 = 1.733, d*f* = 1, *p*=0.001). Furthermore, model #3 contributed to correctly assessing 61.8% of the cases, with high sensitivity (90.2%); however, it presented low specificity (23.9%). The other explanatory variables were not associated with *S. aureus* nasal carriage (*p* > 0.05) (Table 2). Accordingly, male participants had 2.107 (95% CI: 1.268–3.502; *p*=0.004) times the chance of being a carrier of *S. aureus* compared to female participants. Additionally, students had 1.819 (95% CI: 1.105–2.996, *p*=0.019) times the chance of being a carrier of *S. aureus* compared to healthcare workers (Table 2). None of the analyzed variables were associated with MRSA nasal carriage (Table 1).

Few studies analyzing together both cohorts and healthcare workers and students attending a hospital are described in the literature. Hogan et al. [14] reported a low prevalence of *S. aureus* nasal colonization in healthcare workers (10.4%) and in nonmedical students (11.4%) of the University of Madagascar. In contrast to our results, the carriage was higher in female participants and increased with age. Hussein et al. [15] reported a prevalence of 22.5% and 18.7% of *S. aureus* nasal carriage in healthcare workers, and students of a preparatory school that had no contact with a hospital, respectively, in Iraq. None of the variables analyzed (gender, age, and working hours per day or year) were associated with *S. aureus* colonization. Finally, El-Mahdy et al. [18] reported a prevalence of 26.0% of *S. aureus* nasal carriers among both healthcare workers and clinical students in Saudi Arabia. These authors did not evaluate the risk factors associated with colonization.

Previous studies performed with individual cohorts showed that the prevalence of nasal colonization by *S. aureus* and MRSA is highly variable. For instance, reported prevalence of *S. aureus* and MRSA carriers ranged from 12.0 to 39.8% and 0.2 to 22.6%, respectively, among healthcare workers [13, 16, 17, 37, 38], and 15.0 to 40.8% and 0 to 8.0%, respectively, among students [20, 39–41].

In Brazil, few reports about the prevalence of nasal *S. aureus* carriage in healthcare workers and students have been reported. In a study conducted with healthcare workers of the University Hospital of Recife, Pernambuco, a prevalence of 25.7% (52/202) of *S. aureus* carriers was identified, where 5.8% (3/52) were MRSA [13]. In another study, a prevalence of 21.7% (30/138) was observed in nursing students of the Federal University of Piauí, Piauí, and, among the carriers, 23.3% (7/30) were colonized with MRSA [41]. Similar to our results, a high prevalence (102/250, 40.8%) of colonization by *S. aureus* was found among undergraduate biomedical students of the State University of Maringá, in the northwest of Paraná. MRSA was identified in 5.9% (6/102) of colonized students [20].

### 3.3. *S. aureus* Isolated from Healthcare Workers and Students Exhibits Different Antimicrobial Susceptibility Patterns

In our hospital, vancomycin and linezolid are the most used antimicrobials for the treatment of MRSA infections, whereas the others analyzed in this study are used in MSSA infections. The majority of *S. aureus* isolates exhibited susceptibility to most antimicrobials, and this result is consistent with studies reported previously [14], including in Brazil [13, 20, 41]. According to the phenotypic results, six (4.3%) out of 139 isolates were susceptible to all antibacterial agents tested in this study, and all were susceptible to vancomycin. The majority of the isolates were also susceptible to chloramphenicol (135/139, 97.1%), ciprofloxacin (120/139, 86.3%), gentamicin (124/139, 89.2%), linezolid (138/139, 99.3%), rifampicin (138/139, 99.3%), sulfamethoxazole/trimethoprim (123/139, 88.5%), tetracycline (129/139, 92.8%), and tigecycline (138/139, 99.3%). Conversely, a high rate of resistance was observed for penicillin (126/139, 90.6%), erythromycin (93/139, 66.9%), and clindamycin (82/139, 59.0%) (Table 3). Among erythromycin-resistant isolates, 82.8% (77/93), 3.2% (3/93), and 14.0% (13/93) displayed the iMLS_B_, cMLS_B_, and M phenotypes, respectively.

Similar to our results, high resistance rates to beta-lactams antimicrobials, such as ampicillin and penicillin, have been reported to *S. aureus* isolated from nasally colonized individuals from both cohorts [17, 18, 20, 36, 40, 41]. However, the rates of clindamycin/erythromycin resistance may vary according to the geographical region. Low resistance rates were observed in studies from Colombia [39] and Italy [40]. Conversely and consistent with our findings, high rates of resistance to both antimicrobials were detected in Iran [38], China [36], and Brazil [20, 41]. Finally, in some countries, high resistance rates were detected only for erythromycin, including Saudi Arabia [18] and Ethiopia [17].

Herein, *S. aureus* isolates were classified into 38 groups according to the antimicrobial resistance profile (antibiotype) (Table 4). Although a high number of antibiotypes was detected, the majority consisted of a single isolate, and the most frequent profile was coresistance to penicillin, erythromycin, and clindamycin (43/139, 30.9%), followed by resistance to penicillin (30/139, 21.6%). Importantly, 24.5% (34/139) of these isolates were classified as multidrug-resistant, that is, being resistant to three or more antimicrobial classes [42].

**Figure 2:**
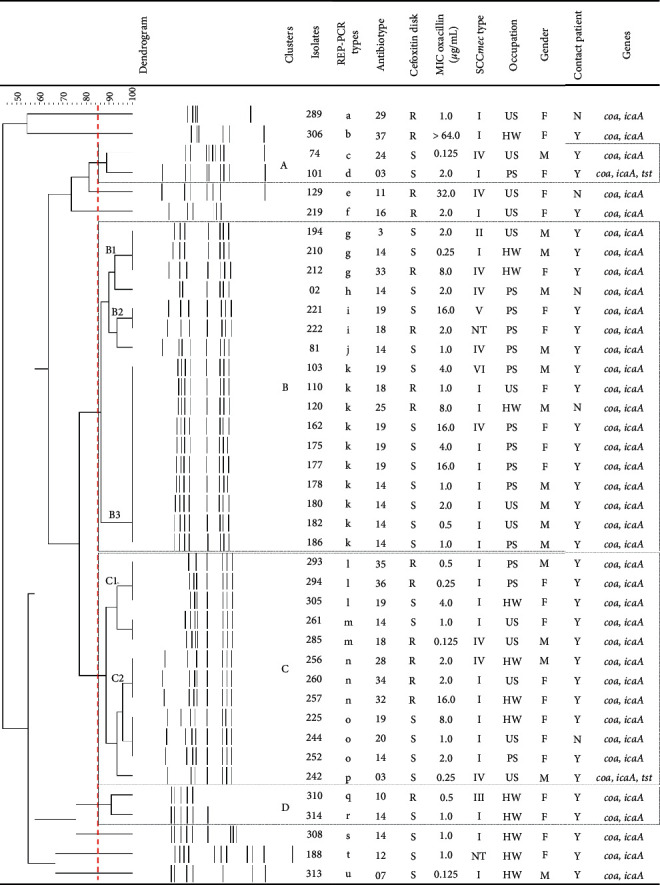
Phenotypic and molecular characteristics of 40 methicillin-resistant *Staphylococcus aureus* (MRSA, *mecA* positive) and descriptive variables of MRSA carriers. The UPGMA banding pattern obtained by REP-PCR based on Dice similarity coefficient showing the genetic relatedness of MRSAs. Vertical dashed line marks the position of similarity coefficient value of 0.85. REP-PCR: repetitive element sequence based-polymerase chain reaction; MIC: minimal inhibitory concentration; R resistant; S susceptible; SCC*mec*: staphylococcal cassette chromosome *mec*; NT: nontypeable; HW: healthcare worker; US: undergraduate student; PS: postgraduate student; F: female; M: male; N: no; Y: yes.

Another important finding of our study is that a high proportion of MRSA isolates (18/40, 45.0%) was not detected by phenotypic methods used for identifying methicillin resistance. Indeed, MRSA isolates (*n* = 40) exhibited different patterns of susceptibility to cefoxitin/oxacillin: (a) 22 (55.0%) exhibited methicillin-resistant phenotype in at least one test; (b) 15 (37.5%) were classified as resistant according to 30 *µ*g cefoxitin disk diffusion assay; (c) 12 (30.0%) displayed oxacillin MIC values ≥4 *µ*g/mL and were classified as resistant; (d) only five isolates exhibited concordant results in both phenotypic methods; (e) 18 (45.0%) were susceptible to methicillin by both phenotypic methods and can be classified as *mecA*-positive oxacillin-susceptible *S. aureus* (OS-MRSA) (Figures 1 and 2).

Currently, OS-MRSA isolated from both infection [7, 43, 44] and nasal colonization [18] has been increasingly reported worldwide. The presence of OS-MRSA carriers and the potential risk of its transmission within the hospital and community are of concern. This bacterium can be misidentified as MSSA by phenotypic methods routinely used in most clinical laboratories, including ours, posing a threat to the treatment of staphylococcal diseases. Indeed, we recently reported the first case of fatal infection caused by an OS-MRSA SCC*mec* type IV that was identified as oxacillin-susceptible by the automated phenotypic method and *E*test (MIC = 0.75 *µ*g/mL) in our hospital [7]. Altogether, these findings corroborate the need for additional tests to accurately distinguish the various methicillin-susceptibility phenotypes of *S. aureus*.

### 3.4. MSSA Isolates Exhibit Higher Virulence Potential than MRSA Isolates

The overall prevalence of genes encoding virulence factors in all *S. aureus* was as follows: *coa*, 100% (139/139); *icaA*, 97.8% (136/139); *tst*, 15.8% (22/139); and *lukS-PV*/*lukF-PV*, 6.5% (9/139). Most isolates (109/139, 78.4%) harbored the *coa* and *icaA* genes; the other isolates harbored the following combination: *coa*, *icaA*, *tst*, (18/139, 12.9%); *coa*, *icaA*, *lukS-PV*/*lukF-PV*, (8/139, 5.8%); *coa*, *tst*, (2/139, 1.4%); *coa*, *icaA*, *tst*, *lukS-PV/lukF-PV*, (1/139, 0.7%) (Table 5).

Not surprisingly, all *S. aureus* harbored the *coa* gene that encodes coagulase, a secreted factor that induces intravascular coagulation, allowing bacterial aggregation in blood and hence promotes its survival and dissemination [45]. Moreover, coagulase activity has been used to differentiate between *S. aureus* (coagulase positive) and those staphylococci historically classified as less pathogenic (coagulase negative) [46]. The gene *icaA* is a component of the *icaABDC* operon that encodes the enzymes involved in the biosynthesis of polysaccharide intercellular adhesin (PIA). This adhesin promotes intercellular aggregation during the maturation stage of PIA-dependent biofilm formation by *S. aureus* [47] and is essential for bacterial virulence in murine model of systemic infection [48].

Notably, the genes *lukS-PV*/*lukF-PV* and *tst* were, respectively, detected exclusively and predominantly in MSSA isolates of our study, and, in general, these isolates exhibited resistance to fewer antimicrobials. Accordingly, the *lukS-PV*/*lukF-PV-*positive isolates displayed the antibiotypes 3, 6, 7, 12, and 14; two *tst*-positive isolates were susceptible to all antimicrobials and the others displayed antibiotypes 1, 3, 6, 8, 17, 20, and 23. Only the antibiotic use was positively associated with *S. aureus* harboring *lukS-PV/lukF-PV* genes (44.4% positive *versus* 15.4% negative) (OR: 4.400, 95% CI: 1.087–17.815, *p*=0.038) and this result remained associated after correction for the other descriptive variables (OR: 4.360, 95% CI: 1.077–17.655, *p*=0.039). No variables were associated with the *tst* gene (data not shown).

The presence of PVL encoding genes is highly variable and can be related to *S. aureus* strain types. Indeed, PVL encoding genes have been primarily associated with community-acquired methicillin-resistant *S. aureus* (CA-MRSA) [49]. On the other hand, *tst* gene has been frequently associated with MSSA strains [50]. However, both genes have also been detected in *S. aureus* exhibiting different characteristics than those of these strains [3, 6, 14, 39, 49].

PVL, a bicomponent *β*-pore-forming toxin, targets the cell membranes of polymorphonuclear leukocytes, monocytes, and macrophages and causes tissue necrosis [51]. Invasive infections caused by PVL-positive *S. aureus* strains have been associated with poor outcome and high mortality rates, regardless of the methicillin-resistance [52, 53]. Importantly, the PVL encoding genes are carried by lysogenic bacteriophages that can be induced by the treatment with various antibacterial agents, including ciproﬂoxacin, imipenem, trimethoprim and/or sulfamethoxazole, and tobramycin [49], thus, facilitating the horizontal transmission among different isolates [51]. Furthermore, it has been known that sub-MIC of beta-lactam antibiotics, which can be found within biofilms, enhances the production of PVL *in vitro* and *in vivo* [54]. Although the clinical significance of this effect is still unknown, these data highlight the importance of the continuous monitoring of the antimicrobial susceptibility and virulence potential of *S. aureus* for the proper management of the infections caused by this bacterium.

TSST-1 is a potent exotoxin of the superantigens family that activates T lymphocytes, resulting in systemic inflammation and shock due to the overproduction of cytokines [55]. Infections caused by *tst*-positive *S. aureus* are also potentially fatal [53] and remain an important cause of morbidity among young people, especially in young women [50]. Although *tst* gene is located in a pathogenicity island, it can also be transferred among different isolates by a helper bacteriophage [56].

The limitation of this analysis is that the presence of virulence-encoding genes was evaluated only by PCR, which does not reflect the expression of the phenotype. However, nasal colonization by *S. aureus* harboring PVL and TSST-1 encoding genes in healthcare workers and students attending the hospital settings may represent a potential source for the emergence of a successful *S. aureus* strain highly adapted in hospital environment due to the silent spread of these virulence markers to multidrug-resistant strains and hence severe and difficult to treat infection in patients. Moreover, both populations may serve as agents of cross-transmission of this strain between the hospital and community individuals.

### 3.5. MRSA Isolates Harboring Different SCCmec and REP-PCR Types Are Grouped into Four Main Clusters

MRSA isolates were distributed into six SCC*mec* types and the type I (25/40, 62.5%) was the most frequent, followed by type IV (9/40, 22.5%). One isolate each (1/40, 2.5%) harbored the SCC*mec* types II, III, V, and VI; two isolates (2/40, 5.0%) were classified as NT (Figure 2). The SCC*mec* types identified herein were also detected in MRSA isolated from different staphylococcal infections in our hospital, where SCC*mec* type II was the most prevalent [3, 6, 7].

Visual observation of bands generated by REP-PCR typing showed a total of 21 banding patterns (named a to u), indicating a relatively high genetic diversity among MRSA isolates. REP-PCR banding patterns were automatically sized and all isolates with the same type were recognized to be identical. The level of similarity between the REP-PCR fingerprinting of the MRSA isolates ranged from 44.1 to 100%. Clusters analysis, by using a cutoff value of 85% [3], revealed that the majority of MRSA isolates were distributed into four groups (named as A to D clusters). Seven isolates exhibited unique fingerprinting patterns. Clusters A and D harbored two isolates each of the different REP-PCR and SCC*mec* types. Cluster B consisted of 17 isolates, comprising five REP-PCR (g, h, i, j, and k) and six SCC*mec* (I, II, IV, V, VI, and NT) different types, which were grouped in three subclusters (B1, B2, and B3). Finally, cluster C consisted of 12 isolates, comprising five REP-PCR (l, m, n, o, and p) and two SCC*mec* (I and IV) different types, which were grouped in two subclusters (C1 and C2) (Figure 2).

OS-MRSA isolates harbored the SCC*mec* type I (12/18, 66.7%), type IV (4/18, 22.2%), and type II (1/18, 5.6%) and one was NT (1/18, 5.6%). Moreover, most OS-MRSA isolates belonged to clusters B (8/18, 44.4%) and C (4/18, 22.2%). These results may explain the high prevalence of OS-MRSA nasal carriers in our study.

## 4. Conclusion

This study provided an overview of *S. aureus* nasal carriage among healthcare workers and students attending our hospital. We observed a high prevalence of *S. aureus* exhibiting different antimicrobial resistance profiles, including multidrug-resistant and OS-MRSA isolates; higher prevalence of MRSA harboring the SCC*mec* types I and IV, which is in contrast to our previous studies reporting the prevalence of SCC*mec* type II among MRSA isolated from different infections [3, 6]; and the presence of PVL and TSST-1 encoding genes among these isolates. These data alert us for continuous surveillance of potential reservoirs of *S. aureus* and improvement in the infections control measures for detecting the various methicillin susceptibility phenotypes and virulence potential of *S. aureus*.

## Figures and Tables

**Table 1:** Potential risk factors for nasal carriage with *Staphylococcus aureus* and methicillin-resistant *S. aureus* (*mec*A-positive) isolates among healthcare workers and students attending the University Hospital of Londrina from December 2017 to May 2018.

Variable		*Staphylococcus aureus*			*mecA*		
		Negative *n* (%)	Positive *n* (%)	*p* value	Negative *n* (%)	Positive *n* (%)	*p* value
Gender	Male	40 (21.6)	48 (34.5)	0.010	32 (32.3)	16 (40.0)	0.389
	Female	145 (78.4)	91 (65.5)		67 (67.7)	24 (60.0)	
Age (years)		31.2 (11.9)	28.8 (10.8)	0.054	28.6 (10.2)	29.3 (9.5)	0.719
Antibiotic use	No	151 (81.6)	115 (82.7)	0.796	79 (79.8)	36 (90.0)	0.150
	Yes	34 (18.4)	24 (17.3)		20 (20.2)	4 (10.0)	
Hospitalization	No	176 (95.1)	137 (98.6)	0.092	97 (98.0)	40 (100)	0.365
	Yes	9 (4.9)	2 (1.4)		2 (2.0)	0 (0)	
Occupation	Student	118 (63.8)	103 (74.1)	0.048	76 (76.8)	27 (67.5)	0.259
	Professional	67 (36.2)	36 (25.9)		23 (23.2)	13 (32.5)	
Student	Nursing	24 (20.3)	14 (13.6)	0.596	11 (14.5)	3 (11.1)	0.698
	Pharmacy	19 (16.1)	20 (19.4)		17 (22.4)	3 (11.1)	
	Physiotherapy	10 (8.5)	6 (5.8)		4 (5.3)	2 (7.4)	
	Medicine	17 (14.4)	18 (17.5)		13 (17.1)	5 (18.5)	
	Postgraduate	48 (40.7)	45 (43.7)		31 (40.8)	14 (51.9)	
Patient contact	No	51 (27.6)	28 (20.1)	0.124	23 (23.2)	5 (12.5)	0.153
	Yes	134 (72.4)	111 (79.9)		76 (76.8)	35 (87.5)	

The continuous variables were expressed as mean and standard deviation (SD) and analyzed with Student's *t*-test; the categorical variables were expressed as number (*n*) and percentage and analyzed with *χ*^2^ or Fisher exact test.

**Table 2:** Bivariate logistic regression analyses of descriptive variables associated with *Staphylococcus aureus* nasal carriage among healthcare workers and students attending the University Hospital of Londrina from December 2017 to May 2018.

Model	Explanatory variable	*Staphylococcus aureus*	
		*p* value	OR (95% CI)
#1	Gender (male)	0.004	2.107 (1.268–3.502)
	Occupation (student)	0.019	1.819 (1.105–2.996)

#2	Gender (male)	0.003	2.182 (1.309–3.665)
	Age	0.021	0.975 (0.955–0.996)
	Occupation (student)	0.537	—
	Antibiotic use	0.850	—
	Hospitalization	0.251	—
	Patient contact	0.147	—

#3	Gender (male) occupation (student)	0.001	2.898 (1.553–5.410)
	Age	0.218	—
	Antibiotic use	0.961	—
	Hospitalization	0.215	—
	Patient contact	0.145	—

OR: odds ratio; CI: confidence interval.

**Table 3:** Antimicrobial susceptibility of 139 *Staphylococcus aureus* isolated from nasal swab of healthcare workers and students attending the University Hospital of Londrina in southern Brazil.

Antimicrobial	MSSA *n* = 99 (%)			MRSA *n* = 40 (%)		
	S	I	R	S	I	R
Penicillin	13 (13.1)	—	86 (86.9)	—	—	40 (100.0)
Cefoxitin	99 (100.0)	—	—	25 (62.5)	—	15 (37.5)
Oxacillin	99 (100.0)	—	—	28 (70.0)	—	12 (30.0)
Chloramphenicol	97 (98.0)	—	2 (2.0)	38 (95.0)	1 (2.5)	1 (2.5)
Ciprofloxacin	90 (90.9)	3 (3.0)	6 (6.1)	30 (75.0)	6 (15.0)	4 (10.0)
Clindamycin	47 (47.5)	2 (2.0)	50 (50.5)	8 (30.0)		32 (80.0)
Erythromycin	34 (34.3)	7 (7.1)	58 (58.6)	4 (10.0)	1 (2.5)	35 (87.5)
Gentamicin	91 (91.9)	1 (1.0)	7 (7.1)	33 (82.5)	2 (5.0)	5 (12.5)
Linezolid	99 (100.0)	—	—	39 (97.5)	—	1 (2.5)
Rifampicin	98 (99.0)	—	1 (1.0)	40 (100.0)	—	—
Sulfamethoxazole/Trimethoprim	87 (87.9)	1 (1.0)	11 (11.1)	36 (90.0)	1 (2.5)	3 (7.5)
Tetracycline	93 (93.9)	1 (1.0)	5 (5.1)	36 (90.0)	1 (2.5)	3 (7.5)
Tigecycline	98 (99.0)	—	1 (1.0)	40 (100.0)	—	—
Vancomycin	99 (100.0)	—		40 (100.0)	—	—

Antimicrobial susceptibility was determined by disk diffusion, except oxacillin and vancomycin that were determined by broth microdilution assay [23] and the agar-screen test [25], respectively. Disk diffusion and microdilution results were interpreted as recommended by CLSI [23] except tigecycline that was interpreted according to the EUCAST [24]. —: not detected; S: susceptible; I: intermediate; R: resistant.

**Table 4:** Distribution of antimicrobial resistance profiles of methicillin-susceptible and methicillin-resistant *Staphylococcus aureus* isolated from the anterior nares of healthcare workers and students attending the University Hospital of Londrina in southern Brazil.

Group	Antimicrobial resistance profile	Number of isolates		
		MSSA, *n* = 93	MRSA, *n* = 40	Total, ^*∗*^*N* = 139 (%)
1	STX	1	—	1 (0.7)
2	TE	1	—	1 (0.7)
3	P	27	3	30 (21.6)
4	P, CLO	1	—	1 (0.7)
5	P, CN	1	—	1 (0.7)
6	P, STX	2	—	2 (1.4)
7	P, E	5	1	6 (4.3)
8	P, DA	1	—	1 (0.7)
9	E, DA	5	—	5 (3.6)
10	P, E, FOX	—	1	1 (0.7)
11	P, FOX, OX	—	1	1 (0.7)
12	P, E, STX	1	1	2 (1.4)
13	P, E, CIP	1	—	1 (0.7)
14	P, E, DA	32	11	43 (30.9)
15	P, E, CN	1	—	1 (0.7)
16	P, DA, FOX	—	1	1 (0.7)
17	P, CIP, RD	1	—	1 (0.7)
18	P, E, DA, FOX	—	3	3 (2.2)
19	P, E, DA, OX	—	7	7 (5.0)
20	P, E, DA, STX	3	1	4 (2.9)
21	P, E, DA, CIP	2	—	2 (1.4)
22	P, E, DA, CN	2	—	2 (1.4)
23	P, E, DA, TE	1	—	1 (0.7)
24	P, E, STX, TE	—	1	1 (0.7)
25	P, E, DA, FOX, OX	—	1	1 (0.7)
26	P, E, DA, STX, TGC	1	—	1 (0.7)
27	P, E, DA, CN, TE	1	—	1 (0.7)
28	P, E, DA, FOX, CIP	—	1	1 (0.7)
29	P, E, DA, FOX, CN	—	1	1 (0.7)
30	P, E, STX, CIP, TE	1	—	1 (0.7)
31	P, E, DA, STX, CN	1	—	1 (0.7)
32	P, E, DA, FOX, OX, CN	—	1	1 (0.7)
33	P, E, DA, FOX, OX, LZD	—	1	1 (0.7)
34	P, E, DA, FOX, CIP, CN	—	1	1 (0.7)
35	P, E, DA, FOX, CN, TE	—	1	1 (0.7)
36	P, E, DA, FOX, CIP, CN, TE	—	1	1 (0.7)
37	P, E, DA, FOX, OX, CIP, CLO	—	1	1 (0.7)
38	P, E, DA, STX, CN, CIP, CLO, TE	1	—	1 (0.7)

^*∗*^Six isolates were susceptible to all antimicrobial tested. P: penicillin (10 U); FOX: cefoxitin (30 *µ*g); CLO: chloramphenicol (30 *µ*g); CIP: ciprofloxacin (5 *µ*g); DA: clindamycin (2 *µ*g); E: erythromycin (15 *µ*g); CN: gentamicin (10 *µ*g); LZD: linezolid (30 *µ*g); RD: rifampicin (5 *µ*g) STX: sulfamethoxazole/trimethoprim (23.75/1.25 *µ*g); TE: tetracycline (30 *µ*g); TGC: tigecycline (15 *µ*g). —: not detected.

**Table 5:** Distribution of virulence encoding genes profiles in methicillin-susceptible and methicillin-resistant *Staphylococcus aureus* isolated from the anterior nares of healthcare workers and students attending the University Hospital of Londrina in southern Brazil.

Virulence marker	Number of isolates		
	MSSA *n* = 99	MRSA *n* = 40	Total *N* = 139 (%)
*coa, icaA*	71	38	109 (78.4)
*coa, tst*	3	—	3 (2.2)
*coa, icaA, tst*	16	2	18 (12.9)
*coa, icaA, lukS-PV/lukF-PV*	8	—	8 (5.8)
*coa, icaA, tst, lukS-PV/lukF-PV*	1	—	1 (0.7)

*coa*: coagulase; *icaA*: intercellular adhesion *locus* encoding N-acetylglucosaminyltransferase; *tst*: toxic shock syndrome toxin; *lukS-PV* and *lukF-PV*: *β*-pore-forming Panton-Valentine leukocidin. —: not detected.

## Data Availability

The data generated and analyzed in the current study are included in the article. Data are also available from the corresponding author upon reasonable request.

## References

[1] Krismer B., Weidenmaier C., Zipperer A., Peschel A. (2017). The commensal lifestyle of *Staphylococcus aureus* and its interactions with the nasal microbiota. *Nature Reviews Microbiology*.

[2] Lowy F. D. (1998). *Staphylococcus aureus* infections. *New England Journal of Medicine*.

[3] Oliveira C. F. d., Morey A. T., Santos J. P. (2015). Molecular and phenotypic characteristics of methicillin-resistant *Staphylococcus aureus* isolated from hospitalized patients. *The Journal of Infection in Developing Countries*.

[4] Lee A. S., de Lencastre H., Garau J. (2018). Methicillin-resistant *Staphylococcus aureus*. *Nature Reviews Disease Primers*.

[5] Duarte F. C., Danelli T., Ribeiro M. A. G. (2018). Bacteremia causada por *Staphylococcus aureus*: Uma análise de quinze anos da sensibilidade a antimicrobianos em um hospital terciário do Brasil. *Revista de Epidemiologia e Controle de Infecção*.

[6] Duarte F. C., Tavares E. R., Danelli T. (2018). Disseminated Clonal Complex 5 (CC5) methicillin-resistant *Staphylococcus aureus* SCC*mec* type II in a tertiary hospital of Southern Brazil. *Revista do Instituto de Medicina Tropical de São Paulo*.

[7] Duarte F. C., Danelli T., Tavares E. R. (2019). Fatal sepsis caused by *mecA*-positive oxacillin-susceptible *Staphylococcus aureus*: first report in a tertiary hospital of southern Brazil. *Journal of Infection and Chemotherapy*.

[8] Young B. C., Wu C.-H., Gordon N. C. (2017). Severe infections emerge from commensal bacteria by adaptive evolution. *Elife*.

[9] Paling F. P., Wolkewitz M., Bode L. G. M. (2017). *Staphylococcus aureus* colonization at ICU admission as a risk factor for developing *S. aureus* ICU pneumonia. *Clinical Microbiololy and Infection*.

[10] Maayan-Metzger A., Strauss T., Rubin C. (2017). Clinical evaluation of early acquisition of *Staphylococcus aureus* carriage by newborns. *International Journal of Infectious Diseases*.

[11] Wertheim H. F. L., Kleef M. v., Vos M. C., Ott A., Verbrugh H. A., Fokkens W. (2006). Nose picking and nasal carriage of *Staphylococcus aureus*. *Infection Control & Hospital Epidemiology*.

[12] Mody L., Washer L. L., Kaye K. S. (2019). Multidrug-resistant organisms in hospitals: what is on patient hands and in their rooms?. *Clinical Infectious Diseases*.

[13] Silva E. C. B. F. d., Antas M. d. G. C., Neto A. M. B., Rabelo M. A., Melo F. L. d., Maciel M. A. V. (2008). Prevalence and risk factors for *Staphylococcus aureus* in healthcare workers at a University Hospital of Recife-PE. *Brazilian Journal of Infectious Diseases*.

[14] Hogan B., Rakotozandrindrainy R., Al-Emran H. (2016). Prevalence of nasal colonisation by methicillin-sensitive and methicillin-resistant *Staphylococcus aureus* among healthcare workers and students in Madagascar. *BMC Infectious Diseases*.

[15] Hussein N. R., Assafi M. S., Ijaz T. (2017). Methicillin-resistant *Staphylococcus aureus* nasal colonisation amongst healthcare workers in Kurdistan Region, Iraq. *Journal of Global Antimicrobial Resistance*.

[16] Emaneini M., Jabalameli F., Rahdar H., Leeuwen W. B. V., Beigverdi R. (2017). Nasal carriage rate of methicillin resistant *Staphylococcus aureus* among Iranian healthcare workers: a systematic review and meta-analysis. *Revista da Sociedade Brasileira de Medicina Tropical*.

[17] Legese H., Kahsay A. G., Kahsay A. (2018). Nasal carriage, risk factors and antimicrobial susceptibility pattern of methicillin resistant *Staphylococcus aureus* among healthcare workers in Adigrat and Wukro hospitals, Tigray, Northern Ethiopia. *BMC Research Notes*.

[18] El-Mahdy T. S., Al-Agamy M. H., Emara M M. (2018). Complex clonal diversity of *Staphylococcus aureus* nasal colonization among community personnel, healthcare workers, and clinical students in the Eastern Province, Saudi Arabia. *BioMed Research International*.

[19] Conceição T., de Lencastre H., Aires-de-Sousa M. (2017). Carriage of *Staphylococcus aureus* among Portuguese nursing students: a longitudinal cohort study over four years of education. *PLoS One*.

[20] Prates K. A., Torres A. M., Garcia L. B., Ogatta S. F. Y., Cardoso C. L., Tognim M. C. B. (2010). Nasal carriage of methicillin-resistant *Staphylococcus aureus* in university students. *Brazilian Journal of Infectious Diseases*.

[21] Becker K., Skov R. L., Von Eiff C., Jorgensen J. H., Pfaller M. A. (2017). *Staphylococcus*, *Micrococcus*, and other catalase-positive cocci. *Manual of Clinical Microbiology*.

[22] Hirotaki S., Sasaki T., Kuwahara-Arai K., Hiramatsu K. (2011). Rapid and accurate identification of human-associated *Staphylococci* by use of multiplex PCR. *Journal of Clinical Microbiology*.

[23] Clinical and Laboratory Standards Institute (CLSI) (2019). *Performance Standards for Antimicrobial Susceptibility Testing*.

[24] EUCAST. European Committee on Antimicrobial Susceptibility Testing, http://www.eucast.org

[25] Khatib R., Riederer K., Sharma M., Shemes S., Iyer S. P., Szpunar S. (2015). Screening for intermediately vancomycin-susceptible and vancomycin-heteroresistant *Staphylococcus aureus* by use of vancomycin-supplemented brain heart infusion agar biplates: defining growth interpretation criteria based on gold standard confirmation. *Journal of Clinical Microbiology*.

[26] Ausubel F. M., Brent R., Kingston R. E. (1999). *Short Protocols in Molecular Biology*.

[27] Milheiriço C., Oliveira D. C., de Lencastre H. (2007). Update to the multiplex PCR strategy for assignment of *mec* element types in *Staphylococcus aureus*. *Antimicrobial Agents and Chemotherapy*.

[28] Campbell S. J., Deshmukh H. S., Nelson C. L. (2008). Genotypic characteristics of *Staphylococcus aureus* isolates from a multinational trial of complicated skin and skin structure infections. *Journal of Clinical Microbiology*.

[29] Tiwari H. K., Sapkota D., Sen M. R. (2008). Detection of *Staphylococcus aureus* using coagulase (Coa) gene PCR as the gold standard. *Nepal Medical College Journal*.

[30] Del Vecchio V. G., Petroziello J. M., Gress M. J. (1995). Molecular genotyping of methicillin-resistant *Staphylococcus aureus* via fluorophore-enhanced repetitive-sequence PCR. *Journal of Clinical Microbiology*.

[31] van der Zee A., Verbakel H., van Zon J.-C. (1999). Molecular genotyping of *Staphylococcus aureus* strains: comparison of repetitive element sequence-based PCR with various typing methods and isolation of a novel epidemicity marker. *Journal of Clinical Microbiology*.

[32] Sneath P. H. A., Sokal R. (1973). *Numerical Taxonomy: The Principles and Practices of Numerical Classifications*.

[33] van Belkum A., Verkaik N. J., de Vogel C. P. (2009). Reclassification of *Staphylococcus aureus* nasal carriage types. *The Journal of Infectious Diseases*.

[34] Legrand J., Temime L., Lawrence C., Herrmann J. L., Boelle P. Y., Guillemot D. (2015). Occupational determinants of methicillin-resistant *Staphylococcus aureus* colonization among healthcare workers: a longitudinal study in a rehabilitation center. *Infection Control & Hospital Epidemiology*.

[35] Budri P. E., Shore A. C., Coleman D. C. (2018). Observational cross-sectional study of nasal staphylococcal species of medical students of diverse geographical origin, prior to healthcare exposure: prevalence of *SCCmec, fusC, fusB* and the arginine catabolite mobile element (ACME) in the absence of selective antibiotic pressure. *BMJ Open*.

[36] Xie X., Dai X., Ni L. (2018). Molecular epidemiology and virulence characteristics of *Staphylococcus aureus* nasal colonization in medical laboratory staff: comparison between microbiological and non-microbiological laboratories. *BMC Infectious Diseases*.

[37] Dulon M., Peters C., Schablon A. (2014). MRSA carriage among healthcare workers in non-outbreak settings in Europe and the United States: a systematic review. *BMC Infectious Diseases*.

[38] Pourramezan N., Ohadian Moghadam S., Pourmand M. R. (2019). Methicillin-resistant *Staphylococcus aureus* tracking spread among health-care workers and hospitalized patients in critical wards at a university hospital, Tehran, Iran. *New Microbes and New Infections*.

[39] Bettin A., Causil C., Reyes N. (2012). Molecular identification and antimicrobial susceptibility of *Staphylococcus aureus* nasal isolates from medical students in Cartagena, Colombia. *The Brazilian Journal of Infectious Diseases*.

[40] De Giusti M., Marinelli L., Aurigemma C. (2013). Prevalence of *Staphylococcus aureus* colonization and antibiotic susceptibility: a survey among biomedical students. *Public Health*.

[41] Carvalho M. S. M., Andrade D. F. R. d., Sousa Á. F. L. d. (2016). Colonização nasal por *Staphylococcus aureus* entre estudantes de Enfermagem: subsídios para monitorização. *Revista Brasileira de Enfermagem*.

[42] Magiorakos A.-P., Srinivasan A., Carey R. B. (2012). Multidrug-resistant, extensively drug-resistant and pandrug-resistant bacteria: an international expert proposal for interim standard definitions for acquired resistance. *Clinical Microbiology and Infection*.

[43] Andrade-Figueiredo M., Leal-Balbino T. C. (2016). Clonal diversity and epidemiological characteristics of *Staphylococcus aureus*: high prevalence of oxacillin-susceptible *mec*A-positive *Staphylococcus aureus* (OS-MRSA) associated with clinical isolates in Brazil. *BMC Microbiology*.

[44] Proulx M. K., Palace S. G., Gandra S. (2016). Reversion from methicillin susceptibility to methicillin resistance in *Staphylococcus aureus* during treatment of bacteremia. *Journal of Infectious Diseases*.

[45] Tam K., Torres V. J. (2019). *Staphylococcus aureus* secreted toxins and extracellular enzymes. *Microbiology Spectrum*.

[46] Fairbrother R. W. (1940). Coagulase production as a criterion for the classification of the Staphylococci. *The Journal of Pathology and Bacteriology*.

[47] Cramton S. E., Gerke C., Schnell N. F., Nichols W. W., Götz F. (1999). The intercellular adhesion (*ica) locus* is present in *Staphylococcus aureus* and is required for biofilm formation. *Infection and Immunity*.

[48] Kropec A., Maira-Litran T., Jefferson K. K. (2005). Poly-N-acetylglucosamine production in *Staphylococcus aureus* is essential for virulence in murine models of systemic infection. *Infection and Immunity*.

[49] Saeed K., Gould I., Esposito S. (2018). Panton-valentine leukocidin-positive *Staphylococcus aureus*: a position statement from the international society of chemotherapy. *International Journal of Antimicrobial Agents*.

[50] DeVries A. S., Lesher L., Schlievert P. M. (2011). Staphylococcal toxic shock syndrome 2000–2006: epidemiology, clinical features, and molecular characteristics. *PLoS One*.

[51] Kaneko J., Kamio Y. (2004). Bacterial two-component and hetero-heptameric pore-forming cytolytic toxins: structures, pore-forming mechanism, and organization of the genes. *Bioscience, Biotechnology, and Biochemistry*.

[52] Sicot N., Khanafer N., Meyssonnier V. (2013). Methicillin resistance is not a predictor of severity in community-acquired *Staphylococcus aureus* necrotizing pneumonia-results of a prospective observational study. *Clinical Microbiology and Infection*.

[53] Gillet Y., Henry T., Vandenesch F. (2018). Fulminant staphylococcal infections. *Microbiology Spectrum*.

[54] Dumitrescu O., Choudhury P., Boisset S. (2011). *β*-Lactams interfering with PBP1 induce panton-valentine leukocidin expression by triggering sarA and rot global regulators of *Staphylococcus aureus*. *Antimicrobial Agents and Chemotherapy*.

[55] Xu S. X., McCormick J. K. (2012). Staphylococcal superantigens in colonization and disease. *Frontiers in Cellular and Infection Microbiology*.

[56] Novick R. P., Ram G. (2017). Staphylococcal pathogenicity islands-movers and shakers in the genomic firmament. *Current Opinion in Microbiology*.

